# What is the impact of a pre-hospital geriatrician led telephone ‘Silver Triage’ for older people living with frailty?

**DOI:** 10.1007/s41999-023-00796-9

**Published:** 2023-05-23

**Authors:** H. T. Jones, W. Teranaka, P. Hunter, L. Gross, S. Conroy

**Affiliations:** 1 Central and North West London NHS Foundation Trust, London, UK; 2 MRC Unit for Lifelong Health and Ageing, University College London, 5th Floor, 1-19 Torrington Place, London, WC1E 7HB UK; 3 London Ambulance Service, London, UK; 4 North Central London Integrated Care Board, London, UK

**Keywords:** Geriatric assessment, Frailty, Older people, Pre-hospital, Telemedicine

## Abstract

**Purpose:**

Most older people are conveyed to hospital via ambulance, therefore presenting a focus to reduce hospitalisation. North Central London has introduced ‘Silver Triage’, a pre-hospital telephone support scheme where geriatricians support the London Ambulance Service with clinical decision-making.

**Methods:**

Data from the first 14 months was analysed descriptively.

**Results:**

There have been 452 Silver Triage cases (November 2021 to January 2023). 80% resulted in a decision to not convey. The mode clinical frailty scale (CFS) was 6. CFS did not influence conveyance rates. Prior to triage, paramedics thought hospitalisation was not required in 44% of cases (*n* = 72/165). All paramedics surveyed (*n* = 176) would use the service again. Most (66%, *n* = 108/164) felt they learnt something and 16% (*n* = 27/164) reported it changed their decision-making process.

**Conclusion:**

Silver Triage has the potential to improve the care of older people by preventing unnecessary hospitalisation and has been well received by paramedics.

## Key summary points

**Aim:**

To identify the impact of pre-hospital geriatrician led telephone triage on conveyance rates of older people to hospital in North Central London, UK.

**Findings:**

Silver triage led to a significant reduction in conveyance rates of people living with frailty to hospital and was well received by paramedics.

**Message:**

Geriatricians and paramedics can work together to identify older people living with frailty who might benefit from care in the community over conveyance to hospital.

## Introduction

The Ageing Well programme within the NHS Long Term Plan promotes older people’s independence and person-centred care aligning with the objective of integrated care systems (ICSs) in integrating health and social care into a unified system [1]. Envisaged outcomes include better care coordination for older people through the creation of more rapid community response teams to prevent admission to and accelerate discharge from hospital. Ambulance services provide a unique system wide, pre-hospital response typically with a single ambulance service operating within an ICS, and as most older people admitted to hospital are conveyed by ambulance, it is logical to explore if frailty-attuned support provided to them can impact patient and service outcomes [2].

North Central London (NCL) ICS is made up of five boroughs: Barnet, Camden, Enfield, Haringey and Islington, which are served by five acute and four community healthcare trusts. Across NCL, there are 227 care homes (5868 beds) with most being based in either Barnet (42%) or Enfield (33%) [3]. Data collected throughout 2018 shows there were 5170 London Ambulance Service (LAS) callouts to NCL care homes with 85% of calls leading to conveyance to hospital and hospitalisation in 52% of cases [3].

**Fig. 1:**
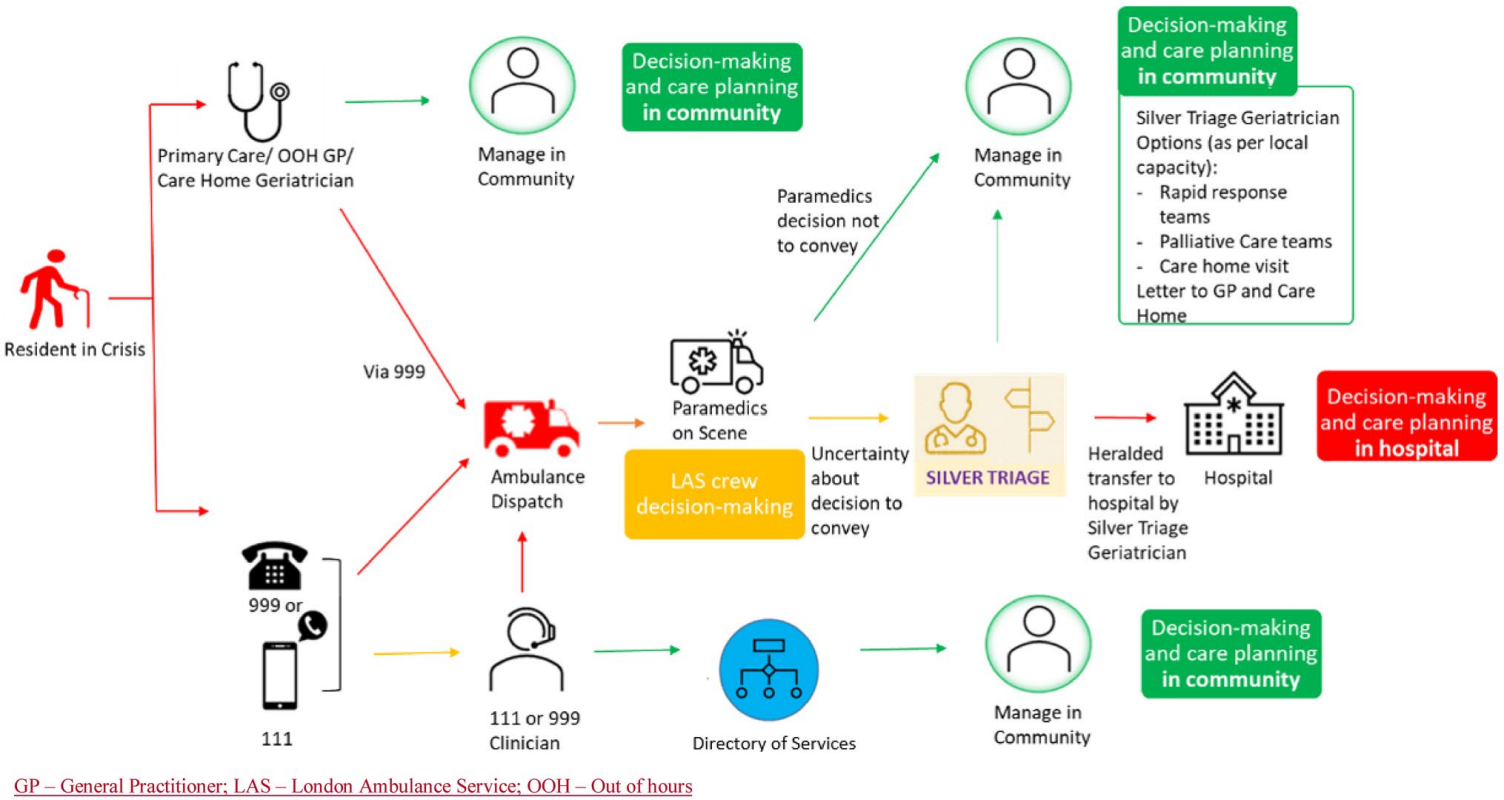
The Silver Triage pathway

NCL ICS invested in a pre-hospital support scheme starting in November 2021, which sees clinicians supporting LAS with clinical decision-making relating to people living with frailty, coined ‘Silver Triage’. The scheme operates 7 days a week from 9:00 to 17.00 and, whilst initially only open to care home residents, it was expanded in January 2023 to people clinical frailty scale (CFS) scores ≥ 6 living in any type of residence [4]. There is a pre-conveyance clinical discussion between paramedics and a consultant geriatrician or emergency physician with geriatrics expertise, accessed via a single telephone number, through which support on decision-making around admission, exploring safer alternatives (e.g. hospital at home) and guiding discussions with patient/families, is provided. Figure 1 situates Silver Triage in the whole system context.

The aim of this work was to assess the initial outcomes and the experiences of paramedics utilising the scheme.

## Methods

A review of the routinely collected data from the first 14 months of Silver Triage was performed [5]. The population included people aged 65 + living with frailty residing in any setting who were attended to by LAS [4]. Data collected included borough of residence, type of residence (own home; assisted living; care home), degree of frailty (CFS) and outcome (conveyance; non-conveyance) [4].

Following Silver Triage, paramedics were sent a survey to explore their opinions collecting closed ended outcomes on whether they felt that patient required hospitalisation prior to Silver Triage, their experience of the process and whether they would use it again [6]. There was also a free text open-ended outcome, exploring how Silver Triage could be improved [6].

Quantitative outcomes were summarised using descriptive statistics such as frequency with corresponding percentages and 95% confidence intervals (CI), whilst the survey outcomes were analysed using thematic analysis with comments coded, grouped and merged to identify themes [7, 8] (Table 1).

**Table 1:** Outcomes of Silver Triage by clinical frailty scale score with 95% confidence intervals

	CFS 5 Living with mild frailty	CFS 6 Living with moderate frailty	CFS 7 Living with severe frailty	CFS 8 Living with very severe frailty	CFS 9 Terminally ill	Total
Proportion of total sample (*n* = 406)	0.09 (0.07–0.12)	0.45 (0.40–0.50)	0.30 (0.26–0.34)	0.14 (0.11–0.17)	0.02 (0.01–0.04)	1.00 (0.99–1.00)
Proportion living in a care home (*n* = 406)	0.62 (0.46–0.76)	0.87 (0.82–0.91)	0.84 (0.77–0.90)	0.98 (0.90–1.00)	1.00 (0.72–1.00)	0.86 (0.82–0.89)
Proportion of total sample post-Silver Triage that remained in the community (*n* = 406)	0.81 (0.66–0.91)	0.79 (0.73–0.84)	0.79 (0.71–0.86)	0.85 (0.74–0.92)	0.80 (0.49–0.94)	0.80 (0.76–0.84)
Proportion of care home residents post-Silver Triage that remained in the community (*n* = 349)	0.83 (0.63–0.93)	0.78 (0.71–0.84)	0.77 (0.68–0.84)	0.85 (0.73–0.92)	0.80 (0.49 -0.94)	0.79 (0.75–0.83)
Proportion of total sample pre-Silver Triage LAS felt did not require hospitalisation (*n* = 165)	0.45 (0.21–0.72)	0.40 (0.30–0.52)	0.42 (0.29–0.56)	0.52 (0.34–0.69)	0.60 (0.23–0.88)	0.44 (0.36–0.51)
Proportion of care home residents pre-Silver Triage LAS felt did not require hospitalisation (*n* = 165)	0.36 (0.15–0.65)	0.32 (0.22–0.43)	0.38 (0.26–0.52)	0.52 (0.34–0.69)	0.60 (0.23–0.88)	0.38 (0.31–0.46)

*CFS* clinical frailty scale,* LAS* London Ambulance Service

## Results

Between November 2021 and January 2023, there were 452 Silver Triage calls. Prior to referral criteria expansion, there was a mean of three calls per day which has now risen to ten. Eleven cases were excluded from analysis due to a CFS of less than 5 and 35 excluded due to no CFS being recorded resulting in a final sample of 406. Information regarding borough was recorded in 55% of cases (*n* = 225), with Barnet (46%) and Enfield (26%) being the most common. Data regarding home setting was available in all cases with 86% of people living in a care home, 2% in assisted living and 12% in their own home. Outcomes regarding conveyance were available in all cases with 80% (95% CI 76–84%) of calls resulting in a decision to not convey. For care home residents (*n* = 349), 79% (95% CI 75–83%) were not conveyed. When comparing pre- and post-expansion data, there was no significant difference in conveyance rates in both the overall sample and the care home resident subgroup. As CFS increased, the percentages of people living in a care home also increased, but CFS increase was not associated with higher conveyance rates. The a priori paramedic decision was recorded in 41% of cases (*n* = 165), with paramedics thinking that only 44% (*n* = 72) could be managed without hospitalisation. CFS did not impact paramedic opinion regarding hospitalisation in general or for care home residents.

The results of the paramedic survey were positive, with all 166 respondents stating they would use Silver Triage again and 73% of respondents (*n* = 165) classifying the service as ‘super quick’ (21% ‘easy’; 3% ‘OK’; 3% ‘bit of a wait’; 1% ‘took ages’). Most found their interaction with the clinician a constructive one with 66% of respondents (*n* = 164) saying the clinician was ‘really helpful and I learnt something’ and 16% saying it was ‘helpful and changed my decision’. Only 3% thought ‘it was ok’, whilst 15% reported it was ‘interesting but did not really change my decision’.

We received free text 102 comments on how Silver Triage could be improved which were grouped into three main themes, each with subthemes. The first theme was ‘improving access to the service’ with subthemes including making it a 24-h service, “Expanding operating times would help… We see a lot of [older] patients … before 9:00”. The other subthemes were expanding into other areas of London and making Silver Triage open to people living in their own homes, the latter of which has already been introduced. The final subtheme was opening the scheme up to emergency ambulance crews (EAC) and emergency medical technicians (EMT) given their increasing role in pre-hospital care, “Give consideration to a robust telephone consultation with paramedic allowing EAC/EMT to refer to Silver Triage”. The second theme was ‘improving information about the service’ with paramedics wanting clearer information about when to use Silver Triage and what information they would be asked for, “I want to know why I am doing something and what they [Silver Triage] can bring to the situation”. The second subtheme was increasing awareness of the scheme in emergency departments, “at handover they didn't really care about us speaking to Silver Triage”. The final theme was ‘improving the delivery of the service’ which covered the subthemes of technological issues, preference for video conferencing and frustrations with pathway breakdown following a decision to opt for community-based care, “After speaking to Silver Triage I was advised to go through the out of hours doctors for a prescription of antibiotics [they] refused to do so … the patient was conveyed to [hospital]”.

## Discussion

Our results suggest that Silver Triage has impacted the care of older people. When compared to the 2018 pre-intervention data, the demographics of our sample were similar with most people living in the boroughs of Barnet and Enfield [3]. However, in our cohort, conveyance rates were much lower at 20% compared to 85% in 2018, suggesting that pre-hospital geriatrician involvement may reduce hospitalisation [9]. In our sample, frailty status did not bias clinician recommendations, as conveyance rates were similar across CFS scores, which is important as whilst CFS is a reliable predictor of outcomes in acute care, it should not direct clinical decision-making [10, 11].

However, our rapid service evaluation is limited by bias and confounding, as paramedics may not have called with cases where they felt the need for hospitalisation was clear-cut. The survey of paramedics is impacted by response and desirability bias as well as missing data limiting conclusions regarding service impact [12]. Prior to recommending expansion of the service, we acknowledge that NCL is not representative as in the UK most older people live in rural or coastal areas [13, 14]. NCL also has a lower of number of older people per consultant geriatrician (29,124) compared to the national average (43,587) [15]. We are also currently unable to report data on the wider pathway such as outcome from Silver Triage like referrals to hospital at home. We have, however, identified a signal associated with this intervention that merits further enquiry. This might involve a mixed method study to further refine the intervention, through incorporating more detailed perspectives on what works, when, why and for whom from a range of key stakeholders as well as a contemporaneous control group. This could lead to a complex intervention that could be tested in a controlled trial, for example, a stepped wedge cluster randomised control trial.

Despite the limitations, there are useful findings here, which are timely given the UK policy context of expanding community and ambulance services announced in January 2023 in response to the NHS pressures, which are likely to be mirrored across Europe as systems adapt to manage the ageing population post-pandemic.

## Conclusion

A pre-hospital telephone triage with paramedics can lead to a non-CFS biased reduction in the number of older people conveyed to hospital. This model has potential to be introduced elsewhere after consideration of local need and resources supporting people living with frailty to receive care in the community.
